# Ecosystem management using livestock: embracing diversity and respecting ecological principles

**DOI:** 10.1093/af/vfac094

**Published:** 2023-04-15

**Authors:** Logan Thompson, Jason Rowntree, Wilhelm Windisch, Sinéad M Waters, Laurence Shalloo, Pablo Manzano

**Affiliations:** Department of Animal Sciences and Industry, Kansas State University, Manhattan, KS, USA; Department of Animal Science, Michigan State University, East Lansing, MI, USA; Technical University of Munich, Liesel Beckmann Straße 2, D-85354 Freising, Germany; Teagasc, Animal and Bioscience Research Department, Grange, Dunsany, Co. Meath, Ireland; Teagasc, Animal and Bioscience Research Department, Grange, Dunsany, Co. Meath, Ireland; Basque Centre for Climate Change (BC3), Leioa, Spain; Ikerbasque — Basque Foundation of Science, Bilbao, Spain

**Keywords:** biomass, circularity, ecology, livestock, pasture, sequestration

**Implications**

- Agricultural land is a scarce resource globally and will continue to encounter challenges to sustainably increase food production in the face of global change. Adaptations that make use of livestock should ideally incorporate agroecological principles (e.g., improved circularity), while limiting feed-food competition. However, they should also remain respectful of the diversity of ecosystem contexts, availability of resources, and the various social and economic needs of local populations.
- Herbivores are a natural constituent of the world’s ecosystems and have played a key role in the last several million years. As the numbers of wild herbivores have greatly decreased, largely due to human action, the maintenance of such roles depends on the practice of adequate livestock management. This is the ecological basis for sustainable livestock.
- Well-managed animals function as an integral and productive part of agricultural systems. Among other outcomes, they can convert massive quantities of nonedible biomass (inevitably arising from pasture systems and from growing plants into human food), recycle plant nutrients back to the land, sequester carbon, improve soil health, and offer many ecosystem services.
- To optimize both environmental impact and food supply, the broad and underutilized diversity that is inherent to livestock systems should be mobilized instead of being suppressed. This diversity can, for instance, be observed in terms of species and breeds, but also in terms of production methods and management strategies.

## Introduction

The animal production sector is facing important challenges in the context of global change. This is especially related to population growth, land erosion, a decrease in biodiversity, wastage of water, depletion of resources, disruption of nutrient cycles and eutrophication, and climate change. Even if livestock agriculture has contributed to these problems, as have other forms of human productivity, it can contribute to the solution, provided it operates within an agroecological framework and environmental boundaries, while still respecting primordial principles of diversity (Leroy et al., 2022). The latter relates not only to the biological variety of livestock options as such, but also to the important heterogeneity within ecosystem types, production and management methods, and local needs and resources. Indeed, livestock products and production systems differ, from intensive to extensive, from arctic to tropical, from highly technological to indigenous, or from being a by-product to being the main focus of the system.

Definitions vary, but there are >40 farmed animal species and >7,000 breeds adaptive to specific local needs and context (FAO, 2021). They produce a vast range of foods and services for humans, from diets that are largely inedible for humans. Only a small share of this bounty of diversity is utilized to its full potential, which makes it a valuable resource pool for solutions (UNFSS, 2021). Turning that biological diversity to the ecological context in which the animals are deployed, combined with a proper set of adapted management strategies, will be of utmost importance. In addition, various innovations have the potential to further open new solution spaces, as is the case for precision livestock farming, genetics, feed, robotics, environmental monitoring, and business models (UNFSS, 2021). Livestock can already provide almost half of our global protein requirements while staying within key planetary boundaries (Van Zanten et al., 2018), and more innovation will increase this share even further (Mottet et al., 2018). But besides bringing in protein of higher quality than when derived from plants, animal-source foods also contain highly bioavailable micronutrients that are often difficult to obtain from crops (see elsewhere in this Special Issue; Leroy et al., 2023).

Below, we will outline the importance of 1) the variability of the ecological context in which livestock systems operate, and how this can both constrain and stimulate the potential of animal production, 2) the need to factor in agro-ecological principles, such as improved circularity and minimized feed-food competition, and 3) the positive ecosystem contributions of well-managed livestock and how these are affected by management strategies.

## The Importance of Ecological  Context and Diversity

The role of livestock in the world’s terrestrial ecosystems has been negatively impacted by the dominant views on “Nature” as landscapes predominantly devoid of human influence (Bond, 2019). As a result, many environmentalists are advocating for an intensification of human activities in ecosystems that have been heavily modified, while completely abandoning human activities in lands suitable for ecological restoration – the so-called “land sparing” approach. Such an approach naturally promotes the abandonment of vast lands used by sustainable livestock management, as they are typically considered to have low production potential and high biodiversity and ecosystem functionality. In recent decades, however, substantial evidence from specialized fields is contesting such views (Manzano-Baena and Salguero-Herrera, 2018).

All continents, except for Antarctica, have been significantly affected by human activities. These regions hosted a significant amount of megafauna, which became extinct outside Africa and South Asia only some thousands of years ago. Naïve animals, not used to people, were easy prey for humans expanding beyond their previous range, which added to technological advances in hunting and climate fluctuations. These extinctions would have had a more dramatic effect on ecosystems than what we see today. But the human activities that followed had a similar action as herbivores on landscapes, namely through the use of fire.

Evidence from Africa shows how megafauna kept landscapes open by efficiently consuming massive quantities of vegetation, especially by seasonal migrations that follow the peaks in plant productivity. These seasonal migrations achieve herbivore densities that are well above the ones observed in temperate protected areas, constrained by surrounding human development. Large herbivore migrations are also assumed to have facilitated the formation of deep organic soils that are now used by some of the most productive crop production systems worldwide. Moreover, elephants are known to tumble down trees and have a particularly important influence in keeping woody vegetation at bay. According to data from national parks where herbivore migrations are still possible, current baseline levels of herbivore densities in Africa are large – and often equivalent to livestock densities under local ranching practices. Such high herbivore levels typified many other parts of the world before the megafauna disappeared, but livestock has kept a high herbivore pressure in the ecosystems (Manzano et al., 2023a). In these contexts, the reduction of ruminants leads to the invasion of deep tap rooted woody species that have deleterious effects on ecological function. Hunter-gatherers use fires to contain woody vegetation and promote grassy biomass, increasing the productivity of the ecosystem and the availability of prey. Pastoralists also use fire to increase fodder availability for livestock, while targeted specialized management utilizes animals such as goats to contain and even revert shrub spread, whereas livestock mobility achieves a high degree of efficiency in plant matter use. Their movement allows them to follow plant growth across vast landscapes, and it thereby also increases the quality of fresh forage intake.

**Figure 1.**
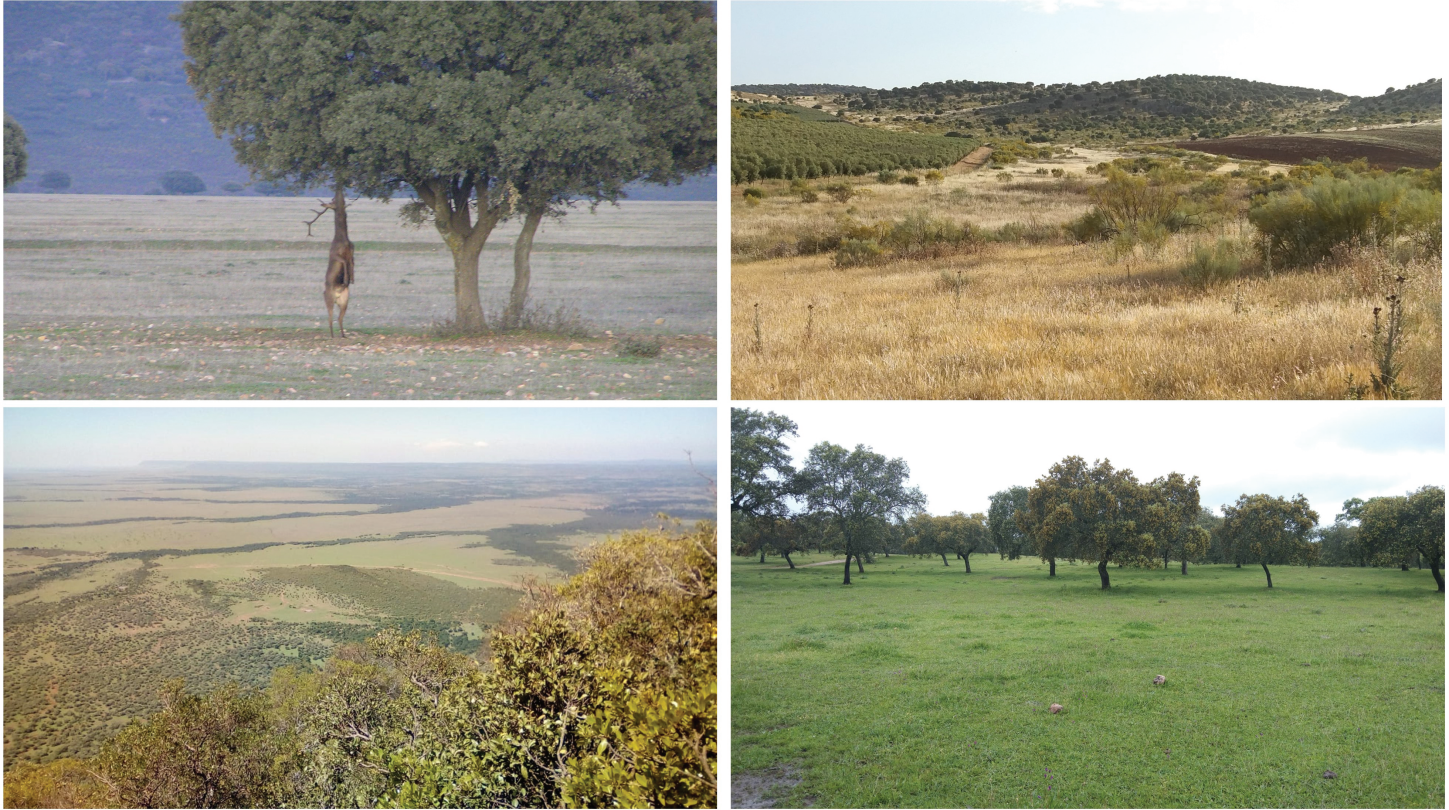
Images of Open Ecosystems (Bond, 2019) dominated by wild herbivores (top left: Cabañeros National Park, Spain; bottom left: Maasai Mara conservancies, Kenya), and of cultural landscapes displaying a similar vegetation structure but that are dominated by domestic herbivores (top right: Conquense Drove Road at Almagro, Spain; bottom right: dehesa in Cordoba municipality, Spain). Pictures’ author: Pablo Manzano.

The resulting landscapes (Figure 1) of all three scenarios – shaped by either megafauna, hunter-gatherers, or livestock keepers – achieves a similar landscape structure of mixed tree groves, shrubs, and open pasture that, except for rainforests, has dominated the planet’s surface since the late Miocene, 12 million years ago. With most plants and dependent animals exposed under the sun during this entire time, biodiversity has adapted to it. Many plant species in biodiverse pastures require considerable amounts of light to survive (Eskelinen et al., 2022), and depend on ecological processes that disturb and contain closed-canopy vegetation.

**Figure 2.**
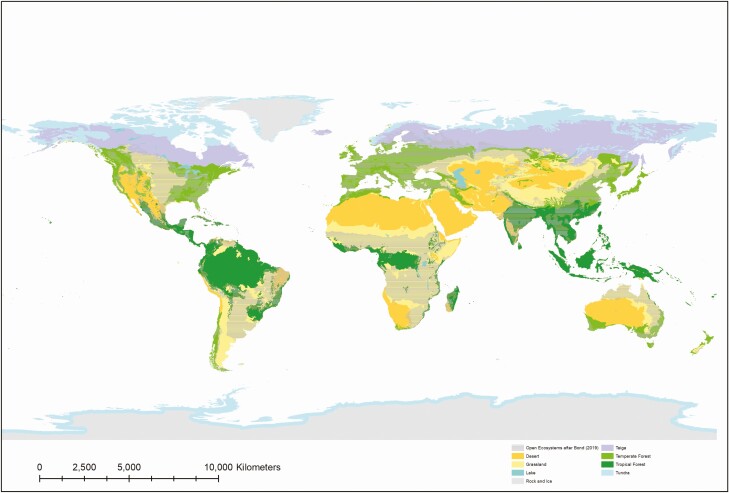
Map of conventionally assumed biomes, defined either as treeless areas or closed canopy forests (Olson et al., 2001), with overlapping areas that display alternative stable states, otherwise defined as Open Ecosystems (Bond, 2019). Map source: Manzano et al. (2023a).

Megafauna is no longer available in Central European, Eastern North American, or East Asian landscapes, so that their abandonment leads to biodiversity losses and the collapse of some high nature value ecosystems, as has been observed in southern Fennoscandia. Once open ecosystems are assumed to be natural (Figure 2), the introduction of extant African and South Asian megafaunal species could be considered as an ecological restoration strategy. But this is no longer viable in contemporary societies because of landscape fragmentation, the presence of infrastructures that render migration impossible, and the high costs of human-wildlife conflict. With adequate management, however, livestock production can provide these important ecosystem services, even if constrained by impacts of human development on the landscape. The necessity of sustainable productive activities to maintain important ecosystem processes, supported by increasing evidence from ecological science (Plieninger et al., 2014; Manzano-Baena and Salguero-Herrera, 2018), reinforces the alternative “land sharing” approach previously advocated for by rural development, human rights, and Indigenous peoples’ advocates.

## The Need to Factor in Agroecology, Circularity, and Feed-food Competition

Traditionally, livestock systems were used to create nutrient rich-food from low opportunity cost feed material. Ruminants were tasked to create food from inedible fodder, whereas monogastrics were fed undesired by-products, such as the residues of potatoes or other types of food-waste. High-productivity livestock systems, however, are increasingly reliant on additional support from feed crops grown on arable land, which engenders feed-food competition (Mottet et al., 2017).

The agroecological potential of livestock primarily relates to the fact that they are able to upcycle copious quantities of nonedible biomass into nutritious foods, while also recycling plant nutrients back to the land, improving soil health, and sequestering carbon (see the next section, below). As such, they are intrinsically connected to sustainable crop agriculture. The purpose of the latter is to generate biomass from plants, of which only a part is suitable for harvest and subsequent production of human food. Even if agricultural innovations have massively increased the volume of biomass produced annually, as well as its subfractions suitable for harvest, most of this output is still nonedible and must ideally be recirculated in view of soil fertility (where animals come in as helpful). Cultivation of wheat and corn, for instance, is highly efficient in the generation of material for harvest compared to nonedible aboveground biomass (e.g., straw), but the ratio remains at 1:1. Other plants cultures show even higher proportions of nonedible biomass (legume seeds, up to 2:1; rape seed, 3:1; sunflower, 4:1). In addition, considerable amounts of nonedible biomass are generated as by-products during food processing, of which the proportion of total inputs ranges between 20% (e.g., intensive milling) and 60% (e.g., production of rape seed oil). While such by-products may still contain human-edible subfractions, they are usually discarded because of technological or economic reasons. Further nonedible biomass arises from inclusion of “green fertilizers” (e.g., use of clover-grass mixtures or alfalfa into the crop rotation systems of organic farming). This practice increases soil fertility but also blocks cultivation of crops at least every fifth year, hence increasing the direct nonedible biomass from arable land by 20% at the expense of human edible food production.

Whereas monogastric animals are particularly useful for the efficient conversion of human-inedible by-products from crops, ruminants also have the capacity to make use of grasslands. The latter not only provides a vast source of nonedible biomass but can often also not be converted into arable land (e.g., due to topographical or climatic reasons). Pasture-based production systems, such as grass-fed beef and dairy cattle, thus convert an inedible material for humans into a consumable form of protein. Often this land is unsuitable for arable farming and therefore this form of meat and milk production has no opportunity for direct food production through cropping. Globally, grasslands represent 70% of the total agricultural area, but even in areas with intensive arable farming, considerable proportions of grasslands can be found (e.g., 30% in Germany).

Summing up arable land and grassland, the ratio of nonhuman edible to human-edible biomass accounts for at least 3:1, provided that no green fertilizers nor other green biomass for feeding purposes is grown on arable land. The ratio observed in practice is at least 4:1 in areas where intensive arable farming is practiced (Wirsenius, 2000; GOALSciences, 2022). Globally, the fact that animals utilize forage and crop residues fits the estimate that 86% of global livestock feed does not compete with human food (Mottet et al., 2017). Obviously, there is still room for improvement within the remaining 14%, in view of further reductions of food-feed competition. It needs to be considered, however, that even for the human-edible part of the feed, animals can function as a buffer for surpluses or for crops that are in principle edible but have been discarded because they did not sufficiently meet quality standards.

Circularity of inevitably occurring, nonedible biomass, and the plant nutrients bound therein (nitrogen, phosphorus) is essential to maintain fertility of agricultural areas. Unless they are left to rot (with often negative consequences on the ecosystem), this may be achieved only through conversion of nonedible biomass into storable organic fertilizers and targeted application to the plants. Two conversion routes are feasible: fermentation in biogas plants ending up in biogas residues or feeding to livestock producing human food as well as manure which is recycled back onto the cropped area in most cases. It is only through livestock that the provision of organic fertilizers will also generate the additional bonus of high-quality human food.

Based on the ecological rationale of circularity, corresponding emissions cannot be attributed to livestock production only. They would also be generated by the inevitably occurring nonedible biomass that would be either left to rot or would be routed to biogas production instead, when not fed to livestock. The same applies to arable land, water, energy, etc., that was consumed during generation of the edible counterpart of the nonedible biomass. Methane released by ruminants during digestion of nonedible feed materials makes a specific difference to the other two pathways of recirculation. Since atmospheric methane is quickly degraded to CO_2_, its long-term effect on global warming is limited, provided ruminant numbers (or their total methane emissions) do not increase (see elsewhere in this Special Issue; Manzano et al., 2023b). On the other hand, conversion of inevitably occurring, nonedible biomass to high quality human food runs without food competition and is most efficient with ruminants.

Taken together, inclusion of livestock into agricultural systems is an efficient driver of circularity. Removing livestock from the system would dissipate the generation of human food from nonedible biomass. Such a system would significantly expand the consumption of resources (land, water, energy) as well as of emissions per nutritional unit (kilocalories, protein, etc.) (van Zanten et al., 2018). Indeed, current high-productivity livestock systems are reliant on intensive production of feed on arable land, thereby charging the environment and climate. But these unfavorable consequences of how intensive livestock production is currently managed, do not justify complete abstinence from livestock. The minimum impact of agricultural production of human food on environment and climate may be found in mixed systems, where livestock production is based on inevitably occurring, nonedible biomass. More circular systems will likely result in smaller and more diverse herd sizes and potentially also a lower output of animal-source foods, even though the latter comes with large uncertainty because some pastoral and agroecological management systems can be remarkably productive (cf., the relatively high stocking rates needed for adaptive grazing). Key to this philosophy is placing livestock where it is most appropriate from a climate, system, and management perspective, thus having an impact on all sustainability indicators.

## Positive Ecosystem Contributions of  Well-managed Livestock and the Link  with Management

Today, much of the discussion on the impact of livestock on the environment commonly focuses on the production of methane, via reticulo-rumen fermentation from ruminant species, leading to a myopic approach to improving the environmental impact of food production. However, the positive impact of livestock on other ecosystem services and as tools to manage and improve the land we rely on for food production can often get overlooked or minimized. One such regulating service is livestock’s impact on soil health and carbon sequestration, particularly in soils that have a legacy of mismanagement. Soil carbon sequestration from livestock production is commonly left out of the greenhouse gas assessments, but in a review of the literature, Cusack et al. (2021) identified soil carbon sequestration as having the largest potential to reduce beef emissions globally, both per unit of product and per unit of land. The potential for carbon sequestration comes via two key mechanisms: 1) restoration of degraded landscapes through the introduction of livestock, and 2) use of adaptive grazing to improve ecological function (Rowntree et al., 2020; Sanderson et al., 2020; Teague and Kreuter, 2020; Grandin, 2022).

Landscape restoration is of particular importance today, considering the legacy of mismanagement found in most soils under agricultural management across the globe (Lal, 2003). These soil losses have occurred primarily in cropping systems and due to overgrazing. Sanderman et al. (2017) estimated that 133 Pg (Petagram) of carbon has been lost in the top 2 m of soil globally due to agriculture. Current and historical farming and ranching practices have aided in this loss of soil carbon due to its growing reliance in past decades on simple annual crop rotations, synthetic inputs, and extractive practices (Lal, 2004). As a result of these practices, soil health and quality have been reduced, along with agricultural outputs from those landscapes. In addition, farmers/ranchers’ reliance on external inputs has increased to keep their operations productive. However, research has indicated that through the introduction of perennial forages, reducing tillage and incorporating livestock, soil carbon can be restored, and overall ecosystem function improved  (Lal, 2004; Rowntree et al., 2020; Teague and Kreuter, 2020). This loss/sequestration of carbon is rarely associated and counted when crop products are compared to livestock systems.

Adaptive grazing management is a nonprescriptive outcome-based approach to grazing management that aims at keeping soil covered, thereby minimizing disturbance (including via overgrazing), minimizing synthetic inputs, and increasing plant diversity. Additionally, management is typically centered on appropriate timing of short-duration, high-intensity grazing events, meanwhile leaving adequate plant residue for plant recovery. The impetus of this management style more closely mimics the natural behavior of grazing animals across landscapes where sporadic, but concentrated and uniform forage utilization is typical. By doing so, plants are allowed adequate time to recover between grazing events, keeping them and their root systems healthy. Additionally, this maintains plants in an active state of regrowth longer, increases solar energy capture, and aids in the cycling of above ground nutrients back into the soil via the physical trampling of plant material and through urine and fecal deposition. Implementation of such management has been observed in numerous studies to improve soil health and water holding capacity, reduce external input requirements, increase soil carbon sequestration, and partially offset the environmental impact of production, amongst numerous other benefits (Teague et al., 2011; Machmuller et al., 2015; Rowntree et al., 2020).

As important as carbon sequestration in grass and rangelands is for the mitigation of environmental damage, it is also paramount that the protection of the carbon that is already stored is safeguarded and that natural ecosystems are kept intact. Globally, permanent grass and rangelands are under threat of conversion into marginal cropping systems or other land uses. In the United States, fueled by ethanol incentives, approximately 3 million ha of grass and rangeland was converted to cropping systems between 2008 and 2012, with the USDA estimating that 960,000 ha of rangeland alone was lost between 2007 and 2015 (Lark et al., 2015; USDA, 2018). These grass and rangelands provide numerous ecosystem services, including wildlife habitat, recreation, and food production, to name a few, and they are often considered to be at a long-term equilibrium for soil carbon (when healthy). Their soils play a critical role in the regulation of global carbon cycling through long-term carbon storage. It is estimated that, globally, grassland and rangeland soils store approximately 20% of soil organic carbon (Conant, 2012). In these arid and semi-arid environments, a key driver to the loss of soil carbon is disturbance from tillage, overgrazing, or urban expansion.

This land conversion serves as a significant carbon source to the atmosphere, along with increasing soil and other nutrient losses (Spawn et al., 2019; Zhang et al., 2021). In the United States, Spawn et al. (2019) estimated that cropland expansion into grasslands resulted in a 38.8 MMTC (million metric tons of CO_2_) yr^−1^ emitted from 2008 to 2012. Similar annual emissions were observed by Yu et al. (2019) from 1980 to 2016. Furthermore, Zhang et al. (2021) simulated the conversion of grassland to crop production in the Midwestern United States and estimated that soil erosion was increased by ~8% annually, and that nitrogen loss increased by ~4%. These losses have impacts beyond anthropogenic emissions, such as sedimentation and eutrophication of waterways that reduce water quality for humans and aquatic ecosystems. The new cropland produced from the land conversion is, typically, of marginal quality as well. The new cropland has a yield deficit of 6.5% of the United States average and has negative impacts on plant and animal biodiversity (Lark et al., 2015). Therefore, while some of these soils may have saturated soil organic carbon stocks, management that protects against degradation/conversion provides significant value by producing high-quality, nutritious human-edible products, protecting the stored carbon from being lost, and protecting natural ecosystems.

## Conclusion

Environmental protection of ecological resources and commercial livestock management are not a contradiction. On the contrary, the one necessitates the other. Commercial livestock management depends on the sustainable provision of ecological resources of water, biodiversity, feeding grounds, and crop land production. At the same time, except for the very few remaining untouched wilderness areas of the world, ecological management towards environmental protection of these resources requires active human management. Livestock are an indispensable instrument in such management to create and sustain the multiple circular flow of materials in the soils, water bodies, and atmosphere.

## About the Author(s)

**Logan Thompson** is an Assistant Professor at Kansas State University in Manhattan, Kansas. He completed his bachelor’s degree at Texas Tech University in 2015. Following this he earned a Masters in beef sustainability at Oklahoma State University in 2017 and a PhD in ruminant nutrition at Michigan State University in 2021. Prior to joining Kansas State University in August of 2022, he served as a post-doctoral fellow at Colorado State University. Logan’s predominate research interest is in measuring greenhouse gas emissions in grazing systems and how grazing management impacts ecosystem function.

**Jason Rowntree** is a professor of Animal Science at Michigan State University where he holds the Charles Stewart Mott Distinguished Professorship for Sustainable Agriculture and is the Co-Director for the newly formed MSU Center for Regenerative Agriculture. Rowntree’s research focus is identifying the metrics and management that reflect ecological improvement in grazing land systems. Rowntree has worked to co-develop, with the Savory Institute, an Ecological Outcome Verification that is now being used on over 2 M acres of grasslands on every continent except Antarctica.

**Wilhelm Windisch** is full professor of Animal Nutrition at the Technical University of Munich (TUM), Germany, since 2010. As an agricultural scientist he finished his doctorate in 1988 and his habilitation in 1995. Prior to his appointment at TUM, he was full professor of Animal Nutrition at the University of Natural Resources and Life Sciences Vienna (BOKU), Austria (2002-2010). He studies nutritional properties of feed materials and principles of biomass transformation in the metabolism of agricultural livestock with particular interest on minerals, functional dietary compounds including antinutritive substances, and the impact of livestock feeding on food quality, food safety, and environment.

**Sinéad Waters** is a Principal Research Scientist at Teagasc, Animal and Bioscience Research Department, Grange, Ireland working mainly in the area of bovine genomics, rumen microbiology and the development of greenhouse gas mitigation strategies. She received a First-Class Honours degree in Biotechnology in 1999, a PhD in gut microbiology in 2003 and was appointed as an adjunct Professor of Agriculture and Climate Change at the Genetics and Biotechnology Laboratory, Ryan Institute, National University of Ireland in 2019. She is the co-chair of the Livestock Research Group of the Global Research Alliance for Agricultural Greenhouse gas emissions (since 2018) and on DG AGRI EU Expert group on methane emissions (since 2021).

**Laurence Shalloo** is a Senior Principle Research Scientist and is Head of the Animal and Grassland Research and Innovation Programme in Teagasc, Ireland. Shalloo’s research interests are in the areas of sustainable livestock production systems. Prior to taking up the role as head of programme, his background was in livestock systems modeling and he has been responsible for the development of models evaluating a range of areas from quantifying the proportion of pasture in a cows’ diet to developing a carbon index for selecting animals for lower overall emissions.

**Pablo Manzano** works as an Ikerbasque Fellow at the Basque Centre for Climate Change in Bilbao, Spain. A rangeland ecologist, he graduated at the Autonomous University of Madrid and has gathered research experience at the FSU Jena, Germany, and University of Helsinki, Finland, plus shorter stays at Landcare Research, Christchurch, New Zealand, and Texas State University, San Marcos, USA. In parallel, he has developed a career in international development cooperation, with experience at several NGOs as well as international organizations such as IUCN, FAO, and the World Bank. His experience has allowed him to perform transdisciplinary research on livestock-based socio-ecosystems.
